# Prevalence and characterisation of exercise-induced laryngeal obstruction in patients with exercise-induced dyspnoea

**DOI:** 10.1017/S0022215123001494

**Published:** 2024-02

**Authors:** Karin Jeppesen, Bahareh Bakhshaie Philipsen, Camilla Slot Mehlum

**Affiliations:** 1 Department of Otorhinolaryngology, Head and Neck Surgery, University Hospital of Southern Denmark, Sønderborg, Denmark; 2 Department of Otorhinolaryngology, Head and Neck Surgery and Audiology, Odense University Hospital, Odense, Denmark

**Keywords:** Airway obstruction, dyspnea, exercise, larynx, laryngeal diseases, laryngoscopy

## Abstract

**Objective:**

The prevalence of exercise-induced laryngeal obstruction is largely unknown. This study aimed to evaluate the prevalence of this condition in a selected study population of patients with exercise-induced dyspnoea.

**Method:**

A retrospective analysis was conducted of demographic data, co-morbidities, medication, symptoms, performance level of sporting activities, continuous laryngoscopy exercise test results and subsequent treatment.

**Results:**

Data from 184 patients were analysed. The overall prevalence of exercise-induced laryngeal obstruction in the study population was 40 per cent, and the highest prevalence was among females aged under 18 years (61 per cent). However, a high prevalence among males aged under 18 years (50 per cent) and among adults regardless of gender (34 per cent) was also found.

**Conclusion:**

The prevalence of exercise-induced laryngeal obstruction is clinically relevant regardless of age and gender. Clinicians are encouraged to consider exercise-induced laryngeal obstruction as a possible diagnosis in patients suffering from exercise-induced respiratory symptoms. No single characteristic that can distinguish exercise-induced laryngeal obstruction from other similar conditions was identified.

## Introduction

Exercise-induced laryngeal obstruction is a condition in which exercise triggers an obstruction in an otherwise non-obstructed larynx,^1^ and may be a differential diagnosis or co-diagnosis of exercise-induced asthma.^2–5^ In order to clarify the diagnosis definitions, in 2013 an international collaborative working committee proposed the inducible laryngeal obstruction nomenclature system.^6,7^ This system facilitates the description of inducing factors (i.e. exercise or irritants) and the level of laryngeal obstructions (i.e. supraglottic, glottic or combined). However, paradoxical vocal fold motion and vocal fold dysfunction are still occasionally used synonymously for exercise-induced laryngeal obstruction.

The prevalence of exercise-induced laryngeal obstruction has mainly been investigated in younger populations^3,4,6^ and groups of athletes.^8,9^ Recently, a multinational study, conducted in Denmark, the UK and the USA, of 1007 patients aged 8–76 years referred on suspicion of exercise-induced laryngeal obstruction, reported an exercise-induced laryngeal obstruction prevalence of 58 per cent.^10^ However, studies describing exercise-induced laryngeal obstruction patients are heterogeneous, and report variable prevalence, test methods and definitions of exercise-induced laryngeal obstruction (Table 1).^2–4,6,8–18^

**Table 1.** Studies describing EILO prevalence

Author	Year, country	Population	Subjects (*n*)	Age of cohort (years)	EILO prevalence & subtypes (%)	% females in EILO group	CLE test?	Definition of EILO	Prevalence of asthma in EILO group (%)
Buchvald *et al*.^11^	2016, Denmark	Children referred with exercise-induced inspiratory symptoms	54	Mean 14, range 9–18	35 (glottic = 11, supraglottic = 61, combined = 28)	61	Yes	CLE test score ≥2 (described by Maat *et al*.^18^)	11
Christensen *et al*.^3^	2011, Denmark	Randomly selected youths (*n* = 556) in Copenhagen were invited; 237 completed questionnaire, 98 participated in CLE test	556 invitees	–	Invitee prevalence = 7.5	–	–	–	–
			98	Median 19, range 14–24	CLE test prevalence = 43 (glottic = 14, supraglottic = 79, combined = 7)	F/M odds ratio 3.41	Yes	Eilomea software	14
Ersson *et al*.^12^	2020, Sweden	367 adolescent athletes in 1st year at sports high school were screened. Randomly selected: 41 patients with exercise-induced dyspnoea & 57 controls	367 screened	Mean ± SD 15.8 ± 0.4	–	–	–	–	–
			34 cases	–	Cases = 15	–	Yes	CLE test score ≥2 (described by Maat *et al*.^18^)	–
			41 controls	–	Controls = 7	–			–
Giraud *et al*.^17^	2023, France	Patients referred for suspicion of EILO in setting of uncontrolled asthma with exercise-related symptoms or exercise-induced inspiratory symptoms	50	Median 16.5, range 14–22	56	80	Yes	CLE test score ≥2 (described by Maat *et al*.^18^)	32
Jansrud Hammer *et al*.^9^	2022, Norway	Athletes competing at national or international level	101	Mean 17.8, range 13–32	72 (supraglottic = 96, glottic = 4)	70	Yes	CLE test score ≥2 (described by Maat *et al*.^18^)	40
Hseu *et al*.^13^	2016, Boston, USA	Patients with complaint of shortness of breath with exercise, with suspicion of paradoxical vocal fold motion disorder	290	Average 14.6	30	77	No	Not reported	10
Johansson *et al*.^4^	2015, Sweden	2309 youths in Uppsala were invited. Randomly selected: 103 adolescents with exercise-induced dyspnoea & 47 controls	2309 screened	Range 12–13	Total = 5.7	49	–	–	–
			99 cases	Median 14.2, range 13–15	Cases = 10.8	63	Yes	CLE test score ≥2 (described by Maat *et al*.^18^)	44
			47 controls	Median 14.2, range 13–15	Controls = 4.8	55			–
Irewall *et al*.^14^	2021, Sweden	Elite cross-country skiers	89	Median 18, range 15–30	27 (supraglottic = 100)	83	Yes	CLE test score ≥2 (described by Maat *et al*.^18^)	42
Nielsen *et al*.^8^	2013, Denmark	Athletes referred with unexplained respiratory symptoms	88	Median 17	35 (glottic = 10, supraglottic = 71, combined = 19)	77	Yes	CLE test score ≥2 (described by Maat *et al*.^18^)	39
Olin *et al*.^15^	2016, Denver, USA	Adolescents & young adults referred for CLE test on suspicion of EILO	71	Mean ± SD 15.0 ± 2, range 12–21	94 (glottic = 21, supraglottic = 3, combined = 76)	66	Yes	4-item visual scoring system (described by Røksund *et al*.^2^)	–
Røksund *et al*.^2^	2009, Norway	Young patients with history of inspiratory distress or stridor during exercise & no evidence of exercise-induced asthma	151 cases	Mean ± SD 16.3 ± 7.3	Cases = 78	72	Yes	4-item visual scoring system (described by Røksund *et al*.^2^)	–
			20 controls	Mean ± SD 22.7 ± 7.8	–	65			–
Shay *et al*.^6^	2020, Texas, USA	Patients aged <26 years with exercise identified as main trigger for breathing symptoms	112	Mean ± SD 15.4 ± 3.0	78 (paradoxical vocal fold motion = 78, supraglottic involvement = 24)	81	No	Not reported	–
Walsted *et al*.^16^	2017, Denmark	Patients referred for investigation of asthma who complained of exercise-related symptoms. Subjects referred with suspicion of EILO were excluded	37	Median 22.5 (IQR 12), range 15–45	22 (glottic = 13, supraglottic = 88)	100	Yes	CLE test score ≥2 (described by Maat *et al*.^18^)	63
Walsted *et al*.^10^	2021, Denmark, UK & New York, USA	Patients referred with possible EILO	1007	Median 25, range 8–76	58 (glottic = 18, supraglottic = 60, combined = 19)	76	Yes	CLE test score ≥2 (described by Maat *et al*.^18^)	36

EILO = exercise-induced laryngeal obstruction; CLE = continuous laryngoscopy exercise; F = female; M = male; SD = standard deviation; IQR = interquartile range

The ‘gold standard’ in diagnosing exercise-induced laryngeal obstruction is the visualisation of the larynx during strenuous exercise. The continuous laryngoscopy exercise test has been validated for this purpose.^19,20^ While the patient exercises on a treadmill or an indoor exercise bike, endoscopy of the larynx is performed to evaluate the anatomical structures during exercise. The patient continues with the activity until laryngeal obstruction symptoms occur or the point of exhaustion is reached.^21^ Normal and pathological continuous laryngoscopy exercise test results are distinguished using arbitrary boundaries ranging from normal glottic and supraglottic abduction to almost complete laryngeal obstruction. Thus, the severity of exercise-induced laryngeal obstruction can be graded using visual or semi-automated grading scores.

Since 2010, continuous laryngoscopy exercise tests have been conducted at the Department of Otorhinolaryngology, Head and Neck Surgery and Audiology, Odense University Hospital, Denmark. Patients are referred for a continuous laryngoscopy exercise test because of exercise-related dyspnoea, cough, stridor and other symptoms indicating a possible exercise-induced laryngeal obstruction diagnosis. According to local referral guidelines, only patients examined initially by a pulmonologist and optimally treated for any pulmonary disease are accepted for the continuous laryngoscopy exercise test.

The primary aim of this study was to evaluate the prevalence of exercise-induced laryngeal obstruction in the study population (regardless of age) with exercise-related respiratory symptoms despite pulmonary testing and appropriate medical treatment. Our secondary aim was to compare patients diagnosed with exercise-induced laryngeal obstruction to the rest of the population in terms of co-morbidity, use of medication, demographical data and patient-reported symptoms, and to assess whether or not any clinical characteristics could indicate a diagnosis of exercise-induced laryngeal obstruction.

## Materials and methods

This retrospective single-centre cohort study included all patients scheduled for a continuous laryngoscopy exercise test at the Department of Otorhinolaryngology, Head and Neck Surgery and Audiology, Odense University Hospital, Denmark, between 1 March 2014 and 28 February 2019. Before the continuous laryngoscopy exercise test, patients completed a questionnaire and an otorhinolaryngologist performed a clinical examination. In January 2016, a visual analogue scale (VAS) score of respiratory symptoms (0, no symptoms; 10, worst imaginable symptoms)^22^ was introduced to the questionnaire. In addition, the ratio of the forced expiratory volume in the first second to the forced vital capacity of the lungs was measured before the continuous laryngoscopy exercise test, and potential airflow obstruction was defined as occurring when this ratio was less than 0.7.^23^

The continuous laryngoscopy exercise test was conducted according to Heimdal *et al*.,^19^ except for the simultaneous ergospirometry test, which was omitted as a pulmonologist had previously examined all patients. A local anaesthetic was applied to the patient's nose and pharynx before a flexible laryngoscope was placed through the nasal cavity into the pharynx, fixed in a specially designed headset and connected to a video camera system with audio recording. The patient's heart rate was measured during the continuous laryngoscopy exercise test to ensure the exercise level reached close to maximum effort. The percentage maximum heart rate reached under exercise was calculated as the measured maximum heart rate divided by 220 minus age in years and multiplied by 100 per cent. Patients continued exercising until general exhaustion or respiratory symptoms prevented further activity.

In this study, exercise-induced laryngeal obstruction was defined as glottic obstruction grade 1–3 (mild, moderate or severe) and/or supraglottic obstruction grade 2–3 (moderate or severe) at maximal effort, in line with the modified continuous laryngoscopy exercise test score proposed by Røksund *et al*.^2^ Obstruction at the supraglottic and glottic levels was scored independently, and exercise-induced laryngeal obstruction was overall classified as ‘supraglottic’, ‘glottic’ or ‘combined’.

Demographic data and information on co-morbidities, medication, symptoms, performance level of sporting activities, continuous laryngoscopy exercise test results and suggested treatments were obtained from the patients’ medical records and a self-reported questionnaire.

Data were analysed in Stata^™^ version 14.2 statistical software. The non-exercise-induced laryngeal obstruction and the exercise-induced laryngeal obstruction patients were compared using the Wilcoxon rank sum test or the *t*-test for continuous variables; the chi-square test was used for categorical variables. *P*-values of less than 0.05 were considered statistically significant.

All procedures involving the participants were conducted in accordance with the ethical standards of Odense University Hospital, Denmark, where the study was conducted. According to Danish legislation, patient consent was not required as exercise-induced laryngeal obstruction tests are part of a standard diagnostic process. The retrospective analysis of data was approved by the Danish Data Protection Agency, the local hospital management team and the legal department at Odense University Hospital (permit number: 19/4948). In addition, the regional ethical committee of Southern Denmark waived the need for approval.

## Results and analysis

A total of 186 (98 per cent) of the 189 patients scheduled for the continuous laryngoscopy exercise test were able to complete the testing. No adverse events or complications were observed. Two patients were excluded from further analysis as they were diagnosed with other specific airway disorders that explained their respiratory complaints, leaving a study population of 184 patients.

Exercise-induced laryngeal obstruction was diagnosed in 74 out of 184 patients (40 per cent), of which 27 out of 46 (59 per cent) were aged under 18 years. Patient characteristics are presented in Table 2. Patients diagnosed with exercise-induced laryngeal obstruction (range, 11–53 years) were statistically significantly younger than non-exercise-induced laryngeal obstruction patients (range, 12–72 years). More patients competing at a national level were diagnosed with exercise-induced laryngeal obstruction (33 per cent) than were not diagnosed with exercise-induced laryngeal obstruction (15 per cent). There were more patients with asthma in the non-exercise-induced laryngeal obstruction group (29 per cent) compared to the exercise-induced laryngeal obstruction group (13 per cent). Twenty patients (of 12 with exercise-induced laryngeal obstruction) used asthma medication despite no self-reported asthma symptoms. Other frequently reported co-morbidities were hypertension, diabetes, gastroesophageal reflux disease in the non-exercise-induced laryngeal obstruction group, and psychiatric disorders and gastroesophageal reflux disease in the exercise-induced laryngeal obstruction group. Medication use reflected these observations, with asthma medication and nasal steroids most frequently used, followed by reflux medication and antihistamines, but differences between the two groups were not statistically significant.

**Table 2.** Patient characteristics

Parameter	Normal findings	EILO findings	Total	*p*-value
*Total population*				
– *n* (% of total)	110 (60)	74 (40)	184 (100)	
Age (median (IQR); years)	31 (19–45)	18 (17–28)	24 (17–41)	<0.001*
Females (*n* (% subpopulation))	75 (68)	58 (78)	133 (72)	0.130
BMI (mean ± SD; kg/m^2^)	24.0 ± 4.6	23.7 ± 4.1	23.9 ± 4.4	0.979
Co-morbidity (*n* (% subpopulation))	*n* = 108	*n* = 70	*n* = 178	
– Asthma	31 (29)	9 (13)	40 (22)	0.013*
– Allergy^†^	40 (40)	22 (34)	62 (38)	0.439
– Other	33 (31)	16 (23)	49 (28)	0.261
– None	52 (48)	46 (66)	98 (55)	0.021*
Medication (*n* (% subpopulation))	*n* = 107	*n* = 73	*n* = 180	
– Asthma medication	39 (36)	21 (29)	60 (33)	0.283
– Other	30 (28)	13 (18)	43 (24)	0.138
– None	48 (45)	44 (60)	92 (51)	0.049*
Performance level of sports (*n* (% subpopulation))	*n* = 94	*n* = 64	*n* = 158	
– Sedentary	14 (15)	4 (6)	18 (11)	0.093
– Community level competitive	66 (70)	39 (61)	105 (66)	0.225
– National level competitive	14 (15)	21 (33)	35 (22)	0.008*
Onset of symptoms (*n* (% subpopulation))	*n* = 89	*n* = 62	*n* = 151	
– <1 year before CLE test	6 (7)	4 (6)	10 (7)	0.944
– 1 to <2 years before CLE test	21 (24)	14 (23)	35 (23)	0.884
– ≥2 years before CLE test	62 (70)	44 (71)	106 (70)	0.863
VAS during symptoms	*n* = 61	*n* = 27	*n* = 88	
– VAS score (mean (range))	7 (2–10)	8 (3–10)	7 (2–10)	0.294
*Children (aged <18 years)*				
– *n* (%)	19 (41)	27 (59)	46 (100)	
Age (median (IQR); years)	14 (13–16)	15 (14–17)	15 (14–17)	0.133
Females (*n* (% subpopulation))	15 (79)	23 (85)	38 (83)	0.583
BMI (mean ± SD; kg/m^2^)	20.2 ± 2.2	21.2 ± 2.4	20.8 ± 2.3	0.184
Co-morbidity (*n* (% subpopulation))	*n* = 19	*n* = 26	*n* = 45	
– Asthma	6 (32)	3 (12)	9 (20)	0.097
– Allergy^‡^	8 (44)	5 (24)	13 (33)	0.173
– Other	2 (11)	4 (16)	6 (13)	0.636
– None	11 (58)	19 (73)	30 (67)	0.286
Medication (*n* (% subpopulation))	*n* = 19	*n* = 27	*n* = 46	
– Asthma medication	8 (42)	10 (37)	18 (39)	0.729
– Other	2 (11)	6 (22)	8 (17)	0.303
– None	10 (53)	13 (48)	23 (50)	0.765
Performance level of sports (*n* (% subpopulation))	*n* = 15	*n* = 25	*n* = 40	
– Sedentary	2 (13)	2 (8)	4 (10)	0.586
– Community level competitive	10 (67)	13 (52)	23 (58)	0.364
– National level competitive	3 (20)	10 (40)	13 (33)	0.191
Onset of symptoms (*n* (% subpopulation))	*n* = 15	*n* = 23	*n* = 38	
– <1 year before CLE test	2 (13)	0 (0)	2 (5)	0.072
– 1 to <2 years before CLE test	5 (33)	6 (26)	11 (29)	0.630
– ≥2 years before CLE test	8 (53)	17 (74)	25 (66)	0.191
VAS during symptoms	*n* = 10	*n* = 10	*n* = 20	
– VAS score (mean (range))	8 (5–10)	7 (3–9)	7 (3–10)	0.758
*Adults (aged* ≥*18 years)*				
– *n* (%)	*n* = 91 (66)	*n* = 47 (34)	*n* = 138 (100)	
Age (median (IQR); years)	39 (24–47)	24 (19–38)	33 (22–45)	<0.001*
Females (*n* (% subpopulation))	60 (66)	35 (74)	95 (69)	0.305
BMI (mean ± SD; kg/m^2^)	24.7 ± 4.6	25.1 ± 4.2	24.8 ± 4.5	0.412
Co-morbidity (*n* (% subpopulation))	*n* = 89	*n* = 44	*n* = 133	
– Asthma	25 (27)	6 (13)	31 (22)	0.064
– Allergy**	32 (40)	17 (40)	49 (40)	0.998
– Other	31 (34)	12 (26)	43 (31)	0.381
– None	41 (46)	27 (61)	68 (51)	0.097
Medication (*n* (% subpopulation))	*n* = 88	*n* = 46	*n* = 134	
– Asthma medication	31 (34)	11 (23)	42 (30)	0.180
– Other	27 (30)	7 (15)	34 (25)	0.047*
– None	38 (43)	31 (67)	69 (51)	0.008*
Performance level of sports (*n* (% subpopulation))	*n* = 79	*n* = 39	*n* = 118	
– Sedentary	12 (15)	2 (5)	14 (12)	0.112
– Community level competitive	56 (71)	26 (67)	82 (69)	0.640
– National level competitive	11 (14)	11 (28)	22 (19)	0.061
Onset of symptoms (*n* (% subpopulation))	*n* = 74	*n* = 39	*n* = 113	
– <1 year before CLE test	4 (5)	4 (10)	8 (7)	0.339
– 1 to <2 years before CLE test	16 (22)	8 (21)	24 (21)	0.891
– ≥2 years before CLE test	54 (73)	27 (61)	81 (72)	0.675
VAS during symptoms	*n* = 51	*n* = 17	*n* = 68	
– VAS score (mean (range))	7 (range 2–10)	8 (range 5–10)	8 (range 2–10)	0.093

*Indicates statistically significant difference between the group with normal findings (non-exercise-induced laryngeal obstruction group) and that with exercise-induced laryngeal obstruction. ^†^Allergy was an independent item in the questionnaire, therefore the percentages are based on the following population sizes: normal findings, *n* = 99; exercise-induced laryngeal obstruction, *n* = 64; and total population, *n* = 163. ^‡^The percentages for allergy are based on the following population sizes: normal findings, *n* = 18; exercise-induced laryngeal obstruction, *n* = 21; and total population, *n* = 39. **The percentages for allergy are based on the following population sizes: normal findings, *n* = 81; exercise-induced laryngeal obstruction, *n* = 43; and total population, *n* = 124. EILO = exercise-induced laryngeal obstruction; IQR = interquartile range; BMI = body mass index; SD = standard deviation; CLE = continuous laryngoscopy exercise; VAS = visual analogue scale

There were no statistically significant differences between non-exercise-induced laryngeal obstruction and exercise-induced laryngeal obstruction patients in the following variables: symptoms arising during exercise (non-exercise-induced laryngeal obstruction: 80 out of 86 patients (93 per cent); exercise-induced laryngeal obstruction: 62 out of 63 patients (98 per cent)), symptom duration of less than 10 minutes (non-exercise-induced laryngeal obstruction: 51 out of 78 patients (65 per cent); exercise-induced laryngeal obstruction: 42 out of 57 patients (74 per cent)), running time on the treadmill (non-exercise-induced laryngeal obstruction: mean (± standard deviation) of 5.2 ± 1.9 minutes; exercise-induced laryngeal obstruction: mean of 5.4 ± 1.5 minutes), running speed on the treadmill (non-exercise-induced laryngeal obstruction: mean of 8.7 ± 2.7 km/hour; exercise-induced laryngeal obstruction: mean of 9.5 ± 2.1 km/hour) or the level of performance measured as a percentage of maximum heart rate. For the non-exercise-induced laryngeal obstruction group, the performance was 95 ± 9.8 per cent of the calculated maximum heart rate and for the exercise-induced laryngeal obstruction group it was 94 ± 9.7 per cent, indicating that patients performed close to their maximum effort during the continuous laryngoscopy exercise test.

As the VAS score of respiratory complaints was introduced during the inclusion period, it was only available for 48 per cent of the population. The mean VAS score was higher in the exercise-induced laryngeal obstruction group than in the non-exercise-induced laryngeal obstruction group, but there were no statistically significant differences.

We found the highest prevalence of exercise-induced laryngeal obstruction (61 per cent) in females aged under 18 years (Table 3). However, we also found a high prevalence among males aged under 18 years (50 per cent) and among adults regardless of gender (34 per cent). Table 4 shows the distributions of exercise-induced laryngeal obstruction subgroups. Thirty-one patients (42 per cent) had supraglottic exercise-induced laryngeal obstruction, 7 patients (9 per cent) had glottic exercise-induced laryngeal obstruction and 36 patients (49 per cent) had combined exercise-induced laryngeal obstruction. In the non-exercise-induced laryngeal obstruction group, 39 patients (35 per cent) were categorised as supraglottic obstruction grade 1, and 71 patients (65 per cent) had no laryngeal obstruction at all during exercise.

**Table 3.** Prevalence of exercise-induced laryngeal obstruction in relation to age and sex

Age	Sex	Normal findings	EILO findings	Total
<18 years	Male	4 (50)	4 (50)	8 (100)
	Female	15 (39)	23 (61)	38 (100)
	All	19 (41)	27 (59)	46 (100)
≥18 years	Male	31 (72)	12 (28)	43 (100)
	Female	60 (63)	35 (37)	95 (100)
	All	91 (66)	47 (34)	138 (100)
Total	All	110 (60)	74 (40)	184 (100)

Data represent numbers (and percentages). EILO = exercise-induced laryngeal obstruction

**Table 4.** Distribution of CLE test scores among patients diagnosed with EILO

Glottic obstruction grade	Supraglottic obstruction grade				Total
	0	1	2	3	
0	–	–	30 (41)	1 (1)	31 (42)
1	3 (4)	15 (20)	14 (19)	1 (1)	33 (45)
2	4 (5)	1 (1)	2 (3)	2 (3)	9 (12)
3	0 (0)	0 (0)	1 (1)	0 (0)	1 (1)
Total	7 (9)	16 (22)	47 (64)	4 (5)	74 (100)

Data represent distribution (numbers (and percentages)) of continuous laryngoscopy exercise scores. CLE = continuous laryngoscopy exercise; EILO = exercise-induced laryngeal obstruction

The most commonly suggested initial treatments for exercise-induced laryngeal obstruction patients (84 per cent) were conservative initiatives such as breathing guidance and/or speech therapy. Twelve of these patients (who had a supraglottic continuous laryngoscopy exercise score of 2 or more) were considered candidates for surgery and further investigation was scheduled. The data regarding surgery results were outside the scope of this study. In patients not diagnosed with exercise-induced laryngeal obstruction, 58 per cent had no further treatment, while others were recommended breathing guidance and/or speech therapy. A total of nine patients (5 per cent) were referred for further investigation on suspicion of other diseases.

## Discussion

The overall prevalence of exercise-induced laryngeal obstruction was 40 per cent in this selected patient group consisting of patients with exercise-induced breathing problems. The highest prevalence of exercise-induced laryngeal obstruction (61 per cent) was in females aged under 18 years, which is similar to other studies,^2,6,24,25^ but we also found a high prevalence of 50 per cent among males aged under 18 years. Among adults, regardless of gender, the prevalence was 34 per cent. Concern has been expressed that focusing on exercise-induced laryngeal obstruction as a diagnosis for young female athletes might bring insufficient attention to this diagnosis in other patients equally affected by exercise-related symptoms.^26^ Our results imply that clinicians must continue to be aware of possible exercise-induced laryngeal obstruction in young female athletes, but also to consider exercise-induced laryngeal obstruction in adults and males with relevant symptoms.

Table 1 shows that previously published studies investigating exercise-induced laryngeal obstruction are heterogeneous, with the reported prevalence of exercise-induced laryngeal obstruction varying from 4.8 per cent^4^ to 94 per cent^15^ in selected populations, based on variable diagnostic methods. In a Danish study by Buchvald *et al*.,^11^ the authors reported a prevalence of exercise-induced laryngeal obstruction of 35 per cent from a selected patient group of children and adolescents aged 9–18 years, referred with exercise-induced respiratory symptoms to a tertiary paediatric pulmonology department. However, in that study, a negative test result was defined as no visible obstruction or objective symptoms, or only mild visible obstruction (score = 1) without subjective complaints. In our study and the international study by Walsted *et al*.,^10^ the diagnosis was solely based on the continuous laryngoscopy exercise test score, regardless of symptoms during the test, which provides a more objective and reproducible evaluation of prevalence.

The heterogeneity of previous studies (Table 1) hampers direct comparison of the prevalence and the severity of exercise-induced laryngeal obstruction, as studies differ in setup, study population, diagnostic method and evaluation of the severity of exercise-induced laryngeal obstruction. The evaluation methods used in continuous laryngoscopy exercise testing are similar, but different cut-offs between normal findings and non-exercise-induced laryngeal obstruction, and mild exercise-induced laryngeal obstruction, can affect the final diagnosis.^2–4,6,8,10,11,13,15,16^ In daily clinical practice, the distinction between normal findings and mild exercise-induced laryngeal obstruction is perhaps less important, as patients with respiratory complaints might benefit from training guidance and/or speech therapy regardless of diagnosis. On the other hand, correct diagnosis is crucial if surgery is to be considered, because this treatment is only recommended for moderate to severe supraglottic obstruction.^27^ In this study, the modified visual continuous laryngoscopy exercise test score, proposed by Røksund *et al*.,^2^ was applied throughout the study period as an easy and quick way to evaluate the video recording while the patient was still present in the examination room.

Several previous studies (Table 1)^3,8,11,16,28^ reported exercise-induced laryngeal obstruction with obstruction at the supraglottic level as the most common subtype of obstruction, whereas our results suggest that exercise-induced laryngeal obstruction with a combined level of obstruction is more common. Most of our patients with combined exercise-induced laryngeal obstruction had grade 1 glottic involvement (Table 4). Differences in the assessment of whether there is no or mild glottic involvement may explain variable results.

In a study by Shay *et al*.,^6^ 78.4 per cent of patients had glottic obstruction and only 23.9 per cent had a supraglottic collapse. The authors did not use the continuous laryngoscopy exercise test but examined patients with fibre laryngoscopy at rest after strenuous exercise, and therefore might have underestimated the prevalence of supraglottic involvement at peak exercise.

The continuous laryngoscopy exercise test, as originally described by Heimdal *et al*.,^19^ is time-consuming, and involves equipment and procedures that may not be available for all clinicians or feasible for subgroups of patients.^6^ In our study, we applied spirometry before the continuous laryngoscopy exercise test, and simultaneous heart rate measurement but not ergospirometry. Flow-volume loops obtained during standard bronchoprovocation testing cannot reliably predict exercise-induced laryngeal obstruction.^3,16,21,29^ Although spirometry is essential for the initial assessment of respiratory complaints, it is not necessary for diagnosing exercise-induced laryngeal obstruction, and could be omitted to simplify the procedure and thus encourage increased continuous laryngoscopy exercise testing.

We found that 12 per cent of the patients with exercise-induced laryngeal obstruction also reported an asthma diagnosis. This is in line with several other studies,^3,11,13,30^ although some studies report up to 25–63 per cent^4,8,10,16,31^ (Table 1). As the symptoms of exercise-induced laryngeal obstruction can mimic exercise-induced bronchoconstriction,^32,33^ clinicians should refer not only patients without asthma but also asthma patients with persistent symptoms despite proper asthma medication for a continuous laryngoscopy exercise test, to diagnose a possible concomitant exercise-induced laryngeal obstruction and prevent the prescription of unnecessary asthma medication.

In our study, 106 patients (70 per cent) had experienced symptoms for at least two years before the continuous laryngoscopy exercise test, suggesting a need for further knowledge about exercise-induced laryngeal obstruction in Denmark to meet patients’ needs for a timely diagnosis.

Personalised treatment for exercise-induced laryngeal obstruction patients is crucial. Local irritants, such as post-nasal secretion in chronic rhinitis or gastric acid in laryngopharyngeal reflux, can contribute to laryngeal sensitivity and induce laryngeal obstruction, but routine medical treatment is not indicated unless patients are otherwise symptomatic.^34,35^ Initially, non-surgical management is recommended;^21^ approximately 75 per cent of exercise-induced laryngeal obstruction patients reported receiving benefits from speech therapy,^36^ laryngeal control therapy lessons,^37^ therapeutic laryngoscopy during exercise^38,39^ or inspiratory muscle training.^40^ In supraglottic obstruction, where non-surgical management is not sufficient, endoscopic laser supraglottoplasty could be considered.^27,39,41^ In our study, 16 per cent of patients were scheduled for follow up to consider endoscopic laser supraglottoplasty.

### Strengths and limitations

The results of this study are probably not directly transferable to the general population because all the included patients suffered from exercise-induced respiratory problems. However, in a hospital setting, the goal is to diagnose and treat patients. The prevalence of exercise-induced laryngeal obstruction in this exact population is therefore important, as they are likely to present with the most severe cases of exercise-induced laryngeal obstruction and thus may be more likely to benefit from specific treatment. Studies that estimate the prevalence of exercise-induced laryngeal obstruction in the general population are still required in order to create a point of comparison for studies on selected populations, as in this present study.

- The ‘gold standard’ for diagnosing exercise-induced laryngeal obstruction is the continuous laryngoscopy exercise test
- The prevalence of exercise-induced laryngeal obstruction is largely unknown
- In this study, the prevalence of exercise-induced laryngeal obstruction in patients experiencing exercise-induced respiratory symptoms was 40 per cent
- The highest prevalence was observed in females aged under 18 years (61 per cent)
- High prevalence was also found in males under 18 years (50 per cent), and in all adults regardless of gender (34 per cent)
- Continuous laryngoscopy exercise testing is recommended for suspected exercise-induced laryngeal obstruction, regardless of patient age or gender

The co-morbidity and use of medication in this study were based on patient-reported questionnaires, which can potentially be imprecise but enable the detection of potential diseases, such as allergies treated with over-the-counter drugs, which would not be detected if the data were obtained from a prescription-related data source.

To our knowledge, this is the first study to investigate the relationship between a self-reported VAS score and disease severity, although the VAS score was first introduced in our population in January 2016 and therefore was available for only 48 per cent of the patients.

## Conclusion

A 40 per cent prevalence of exercise-induced laryngeal obstruction was found in the study population of patients experiencing exercise-induced breathing problems. Patients with exercise-induced laryngeal obstruction were statistically significantly younger, were less likely to have a self-reported asthma diagnosis and their performance level of sports was higher compared to that of the non-exercise-induced laryngeal obstruction group.

Clinicians are encouraged to consider exercise-induced laryngeal obstruction diagnosis in patients suffering from exercise-induced respiratory symptoms, regardless of age or gender, and refer them for continuous laryngoscopy exercise tests. Although certain patient characteristics are more prevalent among patients with exercise-induced laryngeal obstruction, no single characteristic can distinguish exercise-induced laryngeal obstruction from other conditions. Thus, the continuous laryngoscopy exercise test remains the diagnostic gold standard diagnostic test for exercise-induced laryngeal obstruction.
